# Characterisation of Textile Embedded Electrodes for Use in a Neonatal Smart Mattress Electrocardiography System

**DOI:** 10.3390/s21030999

**Published:** 2021-02-02

**Authors:** Henry Dore, Rodrigo Aviles-Espinosa, Zhenhua Luo, Oana Anton, Heike Rabe, Elizabeth Rendon-Morales

**Affiliations:** 1 Robotics and Mechatronics Systems Research Group, School of Engineering and Informatics, University of Sussex, Brighton BN1 9RH, UK; hd255@sussex.ac.uk (H.D.); R.Aviles-Espinosa@sussex.ac.uk (R.A.-E.); 2 School of Water, Energy and Environment, Cranfield University, Bedford MK43 0AL, UK; Z.Luo@cranfield.ac.uk; 3 Academic Department of Paediatrics, Royal Alexandra Children’s Hospital Brighton, Brighton BN2 5BE, UK; Oana.Anton@nhs.net (O.A.); Heike.Rabe@nhs.net (H.R.)

**Keywords:** electrocardiogram, textile, electrode, electric potential sensor, medical devices, wearables

## Abstract

Heart rate monitoring is the predominant quantitative health indicator of a newborn in the delivery room. A rapid and accurate heart rate measurement is vital during the first minutes after birth. Clinical recommendations suggest that electrocardiogram (ECG) monitoring should be widely adopted in the neonatal intensive care unit to reduce infant mortality and improve long term health outcomes in births that require intervention. Novel non-contact electrocardiogram sensors can reduce the time from birth to heart rate reading as well as providing unobtrusive and continuous monitoring during intervention. In this work we report the design and development of a solution to provide high resolution, real time electrocardiogram data to the clinicians within the delivery room using non-contact electric potential sensors embedded in a neonatal intensive care unit mattress. A real-time high-resolution electrocardiogram acquisition solution based on a low power embedded system was developed and textile embedded electrodes were fabricated and characterised. Proof of concept tests were carried out on simulated and human cardiac signals, producing electrocardiograms suitable for the calculation of heart rate having an accuracy within ±1 beat per minute using a test ECG signal, ECG recordings from a human volunteer with a correlation coefficient of ~ 87% proved accurate beat to beat morphology reproduction of the waveform without morphological alterations and a time from application to heart rate display below 6 s. This provides evidence that flexible non-contact textile-based electrodes can be embedded in wearable devices for assisting births through heart rate monitoring and serves as a proof of concept for a complete neonate electrocardiogram monitoring system.

## 1. Introduction

During birth the cardiorespiratory transition of the foetus from intra- to extrauterine life often involves fast and complex decision making to ensure neonatal survival. Approximately 10% of newborns require interventional respiratory support, such as supplemental oxygen or continuous positive airway pressure (CPAP) to safely make the transition where ~1% of these will need more critical resuscitative interventions [1]. Failure to provide these clinical interventions promptly has serious implications for the newborn including increased mortality, cerebral palsy and impaired motor skills.

The predominant quantitative indicator of the health of a newborn is the heart rate (HR). Both the American Academy of Pediatrics [2] and the European Resuscitation Council [3] recommend assessing the newborn’s heart rate within 1 min of delivery. If the measured HR is less than 100 beats per minute (bpm) positive pressure ventilation should be administered and if it is below 60 bpm chest compressions should be initiated [4]. The reliance of clinical professionals on HR data to inform intervention requires rapid and accurate HR acquisition. The most basic form of HR measurement is either done manually by feeling a pulse (palpation) or by listening to the heartbeat using a stethoscope (auscultation). The clinician counts the number of beats in 6 s and multiplies this by 10 to get the heart rate in beats per minute. These methods provide a rapid acquisition time of 7–19 s [5] and are used for the initial HR assessment. A member of clinical staff is required to administer these tests; however, noise, stress and cognitive load can influence measurement. These readings can vary as much as 15 bpm from actual HR where errors in HR determination have been shown to occur in 26–48% of initial assessments [6].

It is critical that neonatal care providers have access to real-time, suitable and accurate diagnostic information to recognize deviations from typical neonate physiology and take the necessary steps to manage scenarios where intervention is necessary.

Clinical and technological advances in the neonatal intensive care unit (NICU) such as the introduction of the APGAR (appearance, pulse, grimace, activity, and respiration) score in the 1950s and the recent widespread use of the pulse oximeter [PO] have been critical in informing interventions and improving infant outcomes [7,8].

The two predominant medical devices used in the delivery room to assess the HR are the pulse oximeter (PO) and the electrocardiogram (ECG). The pulse oximeter measures heart rate using a photodiode-based measurement of blood perfusion and the electrocardiogram is an electrical measurement of heart activity. The silver chloride (AgCl) based ECG is considered the gold standard in the delivery room [9] and has been demonstrated to be both reliable and accurate in assessing HR, as PO has been shown to underestimate the HR compared to ECG struggling to obtain accurate HRs when the newborn suffers from low peripheral blood perfusion or the HR is less than 100 bpm [10].

These devices both necessitate the application of sensors to the newborn and require the attention of a member of the delivery room staff who could otherwise be performing other tasks. Factoring in the time taken to attach the sensors, the device signal acquisition and HR estimation algorithm window length, the average time from birth to HR reading is >70 s for ECG and >110 s for PO [11]. This precludes these devices for use in the initial HR measurement under 1 min. Additionally, there is the need to clean and prepare the newborn’s skin to remove blood or other fluids that affect signal coupling and the application of an electrolytic interface paste which can potentially become sites of infection. Rigid PO sensors and AgCl electrodes can also potentially cause abrasive damage to the delicate newborn skin. The delay in HR acquisition due to sensor application time, combined with the risks associated with AgCl electrodes to fragile newborn skin are both barriers to adoption. Given these drawbacks, alternative dry electrode technologies have been used for reducing the time required for taking a measurement while improving the skin biocompatibility. A study using dry stainless-steel electrodes [12] showed high quality and rapid signal acquisition in newborns after birth; however, the electrodes are rigid and bulky (dimensions of 5 × 2.5 × 1 cm), thus still being a large and intrusive device. More recently novel silver nanowire dry electrodes [13,14] show promise as substitutes for AgCl gel electrodes having a comparable performance to wet AgCl electrodes in terms of ECG signal to noise ratio for heart rate calculation. Fully organic conductive polymer composite (PEDOT:PSS) based dry electrodes [15] have shown to have excellent biocompatibility [16], self-adhesiveness, conformity and have also been shown to record high quality ECG waveforms with reduced noise interference (25 μV compared to 28 μV) compared to AgCl gel electrodes, thus being suitable for ECG based diagnosis. However, both silver nanowire and conductive polymer dry electrodes have not yet been tested in neonates.

Despite evidence that the electrocardiogram provides clear benefits over PO and palpation/auscultation and the recommendations are that ECG monitoring should be adopted in the NICU [17] for newborns requiring resuscitation, this technology has still not been fully developed. In this study we aim to provide a proof of concept system for ECG monitoring based on a full data acquisition and filtering system and two different materials suitable for use as fabric embedded textile-based electrodes for use in the NICU delivery room. The device is capable of recording the HR within 6 s by embedding novel electric potential sensors into a smart mattress.

The structure of the EPS sensor is described elsewhere [18]. Briefly, the electric potential sensor (EPS) is an electrometer-based amplifier insulating electrode that does not require galvanic contact with the body to acquire biopotential signals. Instead it operates with displacement currents and the traditional electrode-skin interface is replaced with a dielectric material. Here, a reference electrode in contact with the patient’s skin is still required for voltage referencing to the EPS sensor

**Figure 1:**
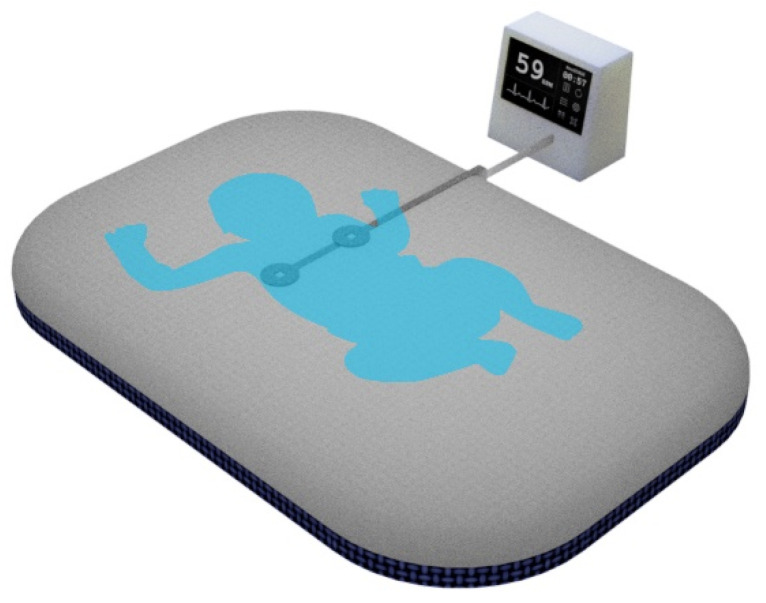
Delivery room mattress with embedded sensors.

Electric potential sensing can be used in place of traditional AgCl electrodes to record high quality ECG signals without the need for electrolytic pastes or location specific sensor application [19]. Previous work using the EPS has shown that it can be used to provide a rapid and reliable ECG reading from a young infant [20]. Textile based conductive electrodes and signal traces can be used to embed these devices in a standard delivery room mattress facilitating the continuous measurement of the HR of a newborn (see Figure 1).

This work aims to investigate the use of textile conductive fabric and screen-printed textile conductive ink electrodes as the reference electrode in an EPS based ECG system. EPS sensors are commonly used with a rigid copper reference electrode. In this paper the three cases (a) copper reference, (b) conductive polymer ink and (c) conductive textile fabric reference electrodes are compared for suitability in recording HR using a custom front end, data acquisition and touchscreen graphical user interface prototype.

## 2. Materials and Methods

**Figure 2:**
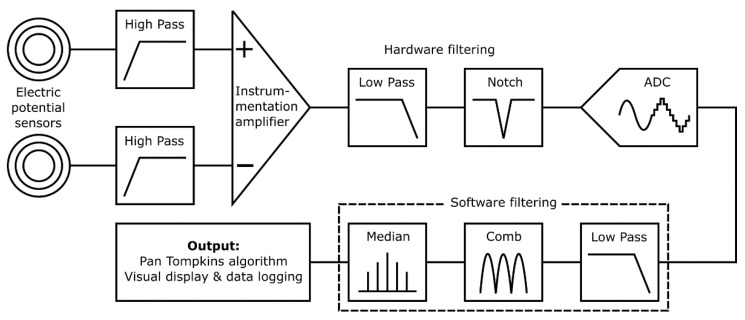
Prototype system signal path with input, filtering and output stages identified.

Figure 2 shows an overview of the prototype system used to characterize the proposed textile-based electrodes. The input stage reads the electrical signal of the heart with a pair of ultra-high input impedance EPS sensors [21] with internal input bias current circuitry and guarding. The voltage outputs from these sensors are sent to the filtering and analogue processing stage for signal conditioning. The resultant cardiac signal is then digitized and additional digital signal processing is performed by an embedded microprocessor. Finally, in the output stage HR calculation is performed by the system and displayed on a 7′′ touchscreen, along with a representation of the signal waveform. The graphical user interface allows for control of the data acquisition and storage methods, as well as filtering and peak detection options.

### 2.1. Input Stage

#### 2.1.1. Sensor Assembly

**Figure 3:**
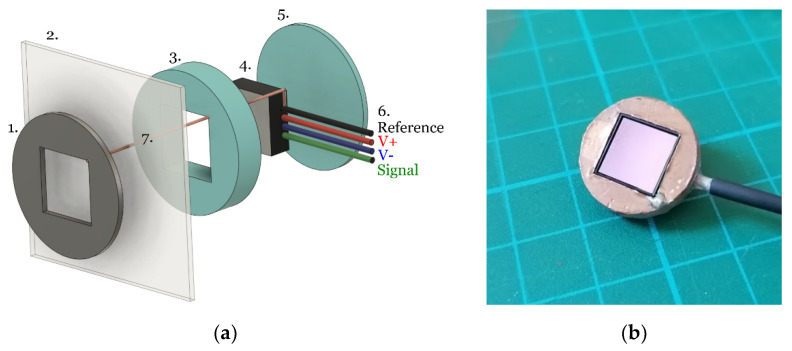
(**a**) Exploded view of sensor: (1) deposited reference electrode material (conductive polymer ink or conductive textile fabric), (2) cotton substrate, (3) and (5) 3D printed housing, (4) EPS ASIC, (6) power and signal connections, (7) single strand copper connection to the sensor ground. (**b**) assembled EPS sensor, with stAandard copper electrode, without the cotton layer attachment.

The EPS devices are application specific integrated circuits (ASICs) contained in a land grid array (LGA) style package measuring 10 × 10 × 2 mm. These are inserted into a 3D printed housing as described in Figure 3.

A single strand of copper wire (diameter ≈ 0.08 mm) was threaded through the cotton and connected to the EPS sensor reference (Figure 3 feature 7) and the patient facing side was covered with an additional layer of the respective electrode material to ensure no copper was present at the skin-electrode interface. The sensor housing was then held in place on the cotton to avoid relative movement between the sensor and reference electrode.

#### 2.1.2. Electrode Fabrication

**Figure 4:**
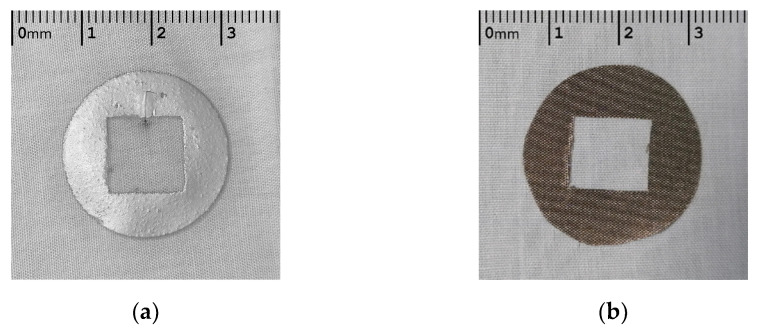
Textile based EPS electrodes, silver conductive ink (**a**) and conductive textile (**b**).

Two materials where considered for embedding the sensors within a NICU mattress in this work. A silver conductive polymer ink (Fabink-TC-C4001) and a self-adhesive conductive textile fabric (MOS TitanRF). Figure 4 shows these two materials deposited on a cotton substrate, with a central cut out of 10 × 10 mm where the EPS electrode was located underneath the cotton.

Considering the inverse linear relationship between electrode size and impedance [22], each electrode had an equal area of 410 mm^2^ to minimise variation in impedance due to electrode geometry. The silver ink was deposited to an average thickness of 0.1 mm and the conductive textile tape was 0.08 mm thick. The conductive polymer ink consists of biocompatible silver flakes suspended in a vinyl resin and perform as well as commercial silver chloride electrodes when screen printed onto fabric [23] and have also been shown to be durable when subjected to washing and abrasion [24].

The silver conductive ink electrodes were screen printed on the cotton substrate and cured at 120 deg C for 10 min to evaporate unwanted solvents and improve conductivity. For the conductive textile fabric, a pattern was printed and the electrode shape was cut out following this. The electrode was then was attached to the cotton substrate using the self-adhesive backing.

The conductive textile fabric is composed of a polyester fibre with metallic copper and nickel composition. These types of commercially available conductive fabrics are shown to be biocompatible and to have low resistance and to keep electrical durability when subjected to repeated washing cycles [25].

**Figure 5:**
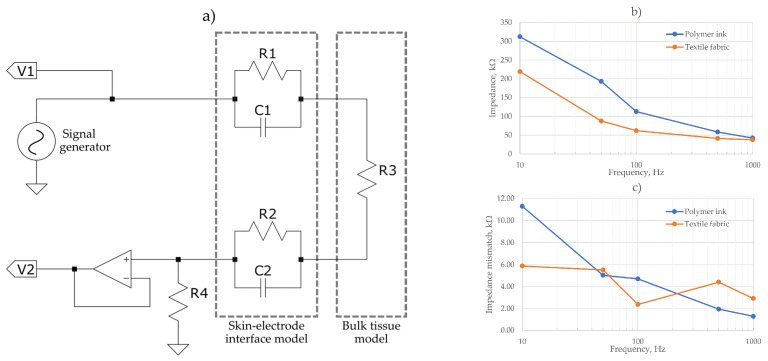
(**a**) Skin-electrode interface and tissue model for impedance measurement setup, (**b**) Measured impedance of the skin electrode interface, (**c**) impedance mismatch between electrode pairs.

The noise induced by the capacitive components of the skin-electrode interface have been shown to dominate the thermal and resistive noise from the electrode alone [26]; therefore, AC impedance measurement of the electrodes was conducted. Figure 5 shows the impedance measurement schematics to assess the skin-electrode interface and tissue model. A Keysight Infiniivision oscilloscope and signal generator was used to calculate electrode impedances at 10, 50, 100, 500 and 1000 Hz. A measurement of impedance at the skin-electrode interface was taken using the IV method described in [27].

Figure 5b shows the calculated impedance from the voltage measurements. The polymer ink electrodes showed marginally an increased impedance across all frequencies than the textile fabric electrodes, but both had a similar impedance profile. The maximum impedance recorded was 300 kΩ at 10 Hz for the polymer ink electrode. Previous works of dry textile ECG electrodes recorded a comparable maximum impedance of 200 kΩ [28] at low frequencies for a polyester/silver textile fabric, dropping off to an average of 50 kΩ for the 500 to 1 kHz range which correlates with the results obtained in Figure 5b.

In Figure 5c the mismatch between the two electrodes of each evaluated material is shown. As it is observed, a maximum mismatch of ~11 kΩ for the polymer ink material and 6 kΩ for the textile fabric was measured, displaying a maximum mismatch of <5% of the total impedance for each frequency. This close impedance matching serves to increase the CMRR and reduce noise.

The relationship between mismatch impedances to common mode rejection ratio (CMRR) is defined using Equations (1) and (2), where ***X*1**, ***X*2**, ***R*1** and ***R*2** correspond to the associated reactance and resistance, respectively:

(1) $$\Delta V=V1_{+}+V1_{-}=V_{cm}\times \left(\frac{X1}{\left(X1+R1\right)}-\frac{X2}{\left(X2+R2\right)}\right)$$

(2) $$\mathrm{CMRR}\left(dB\right)=20\times Log10\left(\frac{1}{\Delta V}\right)$$

Although impedance mismatch between pairs of electrodes could reduce the CMRR and therefore increase the effect of motion artefacts and power line noise on the recordings [29]; the EPS sensor is designed with an external bias circuitry in a way that does not compromise the input impedance of the sensor. Its design includes associated feedback loops providing the functions of guarding, bootstrapping and neutralization to enhance the input impedance, reduce the input capacitance and maintain the electronic stability of the sensor [17,18,19].

### 2.2. Filtering Stage

**Figure 6:**
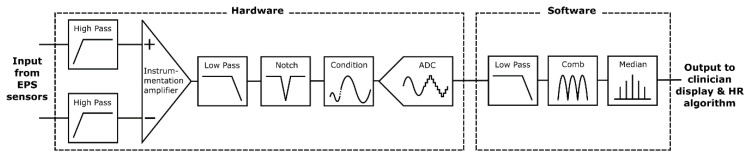
Filtering stage block diagram.

Electrometer based sensors such as the EPS are highly sensitive and susceptible to noise, predominantly 50 Hz power line noise and movement artefacts. The proposed application for this system is a busy NICU where many devices with unknown levels of electromagnetic shielding will be in use and the subject may be in motion due to resuscitation procedures. A dual filtering approach is used employing both hardware and software filters to ensure the 50 Hz noise is sufficiently attenuated to enable an accurate HR measurement. Figure 6 is a simplified diagram outlining the filtering stages implemented.

Initially a pair of passive high pass filters (corner frequency, *f_c_* = 0.5 Hz) remove baseline wander. Next, a subtraction is performed using a precision instrumentation amplifier between the both sensor signals to remove common noise. An active 2nd order low pass (*f_c_* = 200 Hz) and an active 2nd order twin-T form notch filter (*f_c_* = 50 Hz) remove high frequency and power line noise, respectively. Signal conditioning circuitry then scales the signal to ensure the full range of the 12-bit analogue to digital convertor (ADC) is used. This ADC bit depth is sufficient to represent the acquired signal with enough detail to identify the QRS waves required for HR detection.

**Figure 7:**
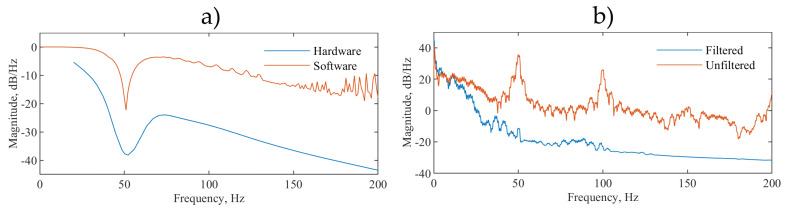
(**a**) Frequency response analysis of hardware and software stages, (**b**) power spectral density showing the combined effect of hardware and software filtering on a noisy test ECG signal injected directly into the front end.

The serial peripheral interface (SPI) output of the ADC is read at 1000 samples per second by a quad-core ARM Cortex-A53 based platform (Raspberry Pi 3 Model A+) which performs additional software filtering with a modified Pan Tompkins algorithm [30] for peak detection and heart rate acquisition. The software filtering consists of a forwards form comb filter (*f_c_* = 50, 100 and 150 Hz), a low pass 20th order finite impulse response filter (*f_c_* = 200 Hz) and a variable median filter for signal smoothing. The software filters are coded in C++ avoiding any external dependencies and to minimise system resource usage. For the filter validation, the EPS sensors were removed. A generated signal employing a NI USB 6009 and LabVIEW biomedical toolkit providing the combined ECG and 50 Hz noise signal was injected to the inverting and noninverting terminals of the instrumentation amplifier. Figure 7a shows the frequency response analysis (FRA) of both the hardware and software stages and Figure 7b shows the power spectral density (PSD):

The majority of the ECG signal power is within the 0.5 to 40 Hz range and the filter corner frequencies are chosen to match the specifications recommended by the American Heart Association, the American College of Cardiology and the Heart Rhythm Society [31] and to minimize uncertainty in the measurement of the R-R peak intervals used to calculate the HR [32]. Figure 7b shows that the peak at 50 Hz which represents the mains electrical hum, in our measurements, the system-induced power line noise is reduced by 40 dB and the subsequent peak at 100 Hz is also not present. This shows the system capacity for filtering 50 Hz and higher harmonic frequency noise that are most common in ECG recordings.

### 2.3. Output Stage

**Figure 8:**
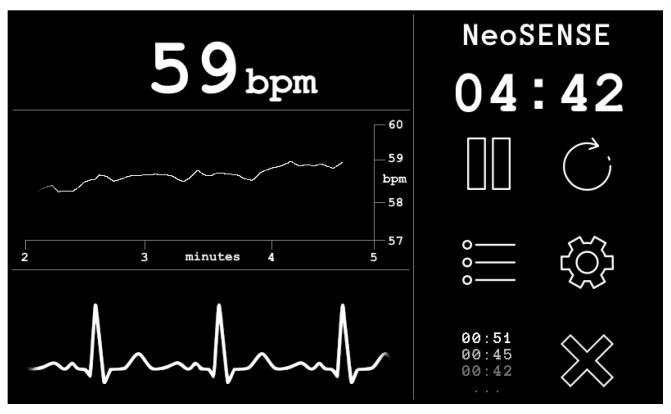
Screenshot of prototype graphical user interface.

A custom graphical user interface (GUI) coded in C++ running on Raspbian Linux OS displays the output of the EPS sensors after the filtering stages on a 7′′ capacitive touch screen display (Official Raspberry Pi 7′′ Touchscreen Display ACGGD070-004-CG-B, resolution 800 × 480 pixels). See Figure 8.

The GUI allows for real time inspection of the cardiac signal and heart rate. Data is stored on the device memory and can be timestamped and reset from within the GUI. The system is portable and can be powered by a standard 5V DC USB power bank, giving a running time of 4 h from a 10,000 mAh battery. Considering IEC 60601 the prototype presented here is classified as an IP (internal power supply) device. The use of a battery DC supply removes the possibility of 50 Hz power line noise contamination of the signal that is often common in devices powered by mains switch mode power supplies that convert AC to DC. Please note that under the classification of patient applied parts the EPS sensors, being non-contact and isolated behind a dielectric material, are not be covered under IEC 60601; however, the reference electrodes are considered as patient applied part floating–surface conductor. In this prototype, the device was designed so that it cannot be operated during the battery charging cycle.

## 3. Experimental Results

The goal of our experimental tests is to assess the suitability of the manufactured textile-based electrodes for use in a full end to end non-contact ECG acquisition system. In order to evaluate these, two ECG recording scenarios were investigated:

1. A simulated cardiac signal employing a neonatal simulation environment;
2. A real cardiac signal from an adult volunteer.

The neonate simulation environment is used to form an initial comparison of ECG reproduction and a baseline comparison of the different electrodes. Then the human volunteer serves to establish a proof of concept for the end to end system. Three electrode configurations were evaluated in each of the two scenarios:

- baseline copper electrodes;
- conductive polymer ink;
- conductive textile fabric.

**Figure 9:**
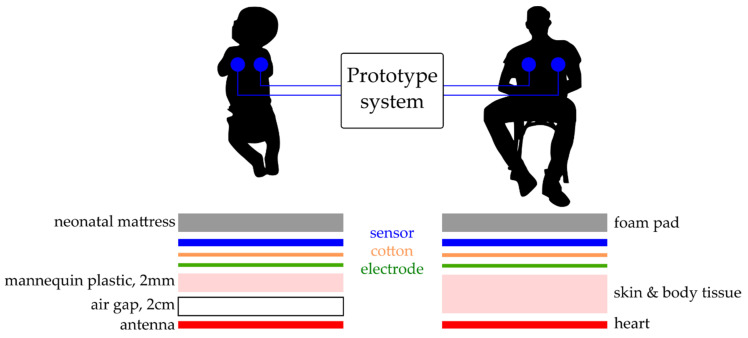
Experimental set up—electrode placement and interference layers for simulated and human ECG readings, left neonate mannequin, right human volunteer.

To simulate signal acquisition of the heartbeat of a neonate a simulated ECG signal was radiated from a premature infant mannequin, with an internal antenna (3 cm^2^). The bio signals were generated in a custom LabVIEW GUI using a USB 6009 National Instruments DAQ with a 16-bit digital to analogue converter (DAC). This test ECG signal was radiated across an air gap of 2 cm and through 2 mm of the electrically insulating plastic of the mannequin having an amplitude of 200 mV peak-to-peak (PP) at 140 bpm. A human ECG was also taken from a male adult volunteer as a proof of concept trial, with the subject sat in a comfortable upright position and the sensor attached to the chest using an additional 5 cm layer of foam padding on the reverse to simulate the delivery room mattress material. Figure 9 shows the position of the sensors and interference layers for both scenarios.

After signal acquisition was carried out, two readings of 60 s were taken for the simulated mannequin signal and five readings of 60 s for the human volunteer. These were exported from the data acquisition system and imported into MatLab for analysis.

### 3.1. Neonatal Simulation Environment

The mannequin providing the simulated ECG signal was used as a control to create a baseline of ECG reproduction regarding the comparison of copper electrodes with the manufactured textile-based electrodes.

**Figure 10:**
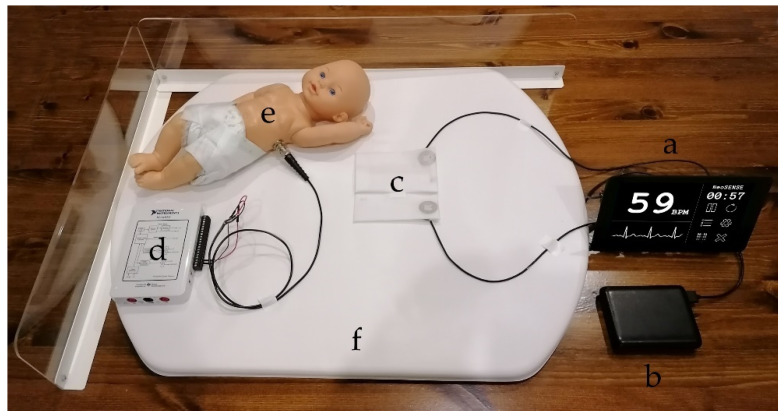
Photograph of prototype system hardware in neonate simulation environment: (a) front end and GUI (Dimensions 195 × 100 × 90mm); (b) USB 5V power bank; (c) EPS sensors and textile based electrodes; (d) digital to analogue converter for test signal generation; (e) neonate mannequin with internal antenna; (f) commercial delivery room mattress.

**Figure 11:**
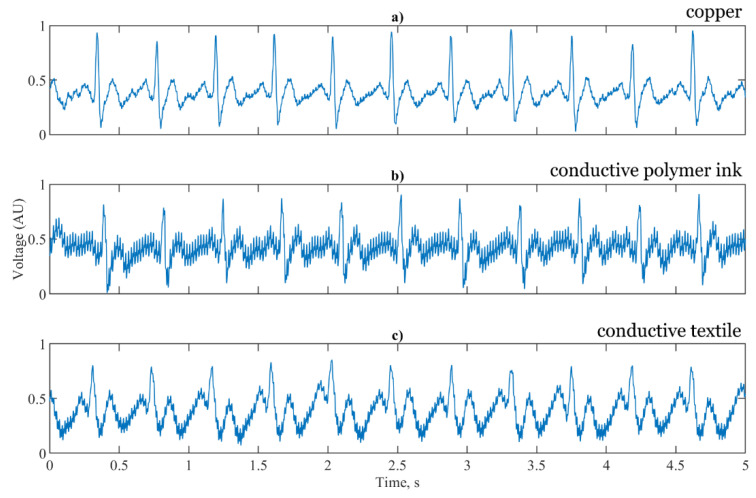
5 s segments of a simulated ECG signal for (**a**) baseline copper electrodes; (**b**) conductive polymer ink; (**c**) conductive textile fabric.

Figure 10 shows the neonatal simulation environment and test setup for the initial signal characterisation providing the simulated ECG signal. The neonate mannequin was placed on top of the electrodes and sensors to perform the recordings. Figure 11 shows a representative sample of 5 s of simulated signal ECG recording.

Figure 11a shows the signal reproduction of the simulated test signal using the copper electrodes, the conductive polymer ink and conductive textile fabric. For accurate HR calculation the R peak of the ECG signal must be clearly visible above the baseline. For each of the three sensor configurations the R peak is prominent and suitable for RR interval calculation. Figure 11b shows the T and P waves of the ECG clearly reproduced, with the same timing as the reference copper electrodes having an increase in the noise components. For the conductive textile fabric electrodes shown in Figure 11c, the waveform is distorted with the T waves elevated, the R peak and subsequent characteristic dip truncated and the amplitude of the P waves increased. High frequency noise is present in the conductive textile fabric electrodes, but at a lower amplitude than the conductive polymer ink electrodes.

**Figure 12:**
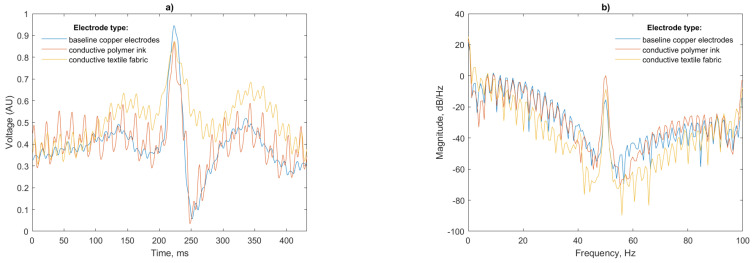
(**a**) Single beat waveform and (**b**) Power spectral density of a simulated ECG simulated from the test mannequin for each electrode configuration.

Analysis of a single beat and power spectral density (PSD) of the recorded signals is presented in Figure 12:

A total of 50 Hz noise is visible for both the polymer ink and conductive textile electrodes. This is more pronounced for the polymer ink electrode. The air gap between the antenna and mannequin body has a capacitance that dominates the total electrode capacitance in the series connection [33] with the insulating layer of the EPS sensor, so any noise in this gap will be amplified in the final signal.

Data from the simulated cardiac signal in a neonatal simulation environment was processed using the Pan Tompkins algorithm for HR calculation and a mean RR interval and average bpm was calculated. Considering the precise bpm of the simulated signal, these results can then be used as a measure of the accuracy of the sensor acquisition and ECG recording stages.

The mean RR intervals as shown in Table 1 are within 0.5% of the expected RR interval of 432 ms for a heart rate of 140 bpm, the heart rate for the normal neonate being between 120 to 160 bpm [2]. The average bpm recorded by the system are with 1 bpm of the simulated signal, which compares favourably with pulse oximeters which commonly have a stated accuracy of ±2 bpm. An additional test signal at 60 bpm confirmed the suitability of the device for recording low heart rates and showed RR intervals within 0.4% of the expected RR interval of 1000 ms and an average within ±0.2 bpm.

### 3.2. Proof of Concept with Human Volunteer

The prototype system was then used to characterize a human cardiac signal. The weight of the heart of an infant is ~14 times smaller than that of an adult [34], resulting in a reduction of the ECG signal amplitude falling within the micro to millivolts range. Nevertheless, EPS sensors have proved to record high quality ECGs from a young infant [20].

**Figure 13:**
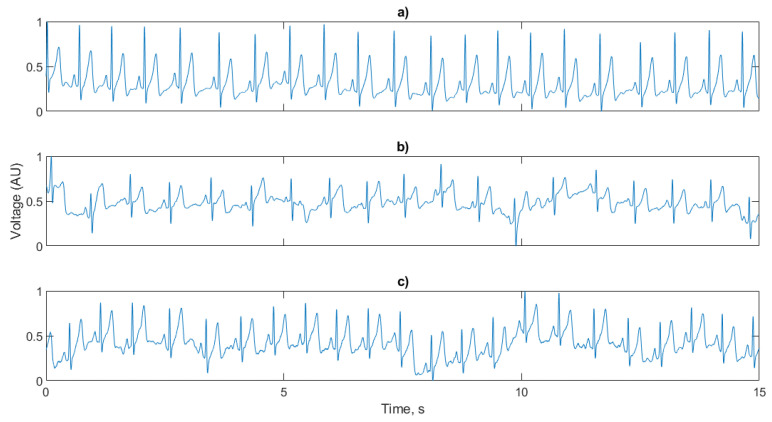
15 s segments of a human signal ECG signal recorded with (**a**) baseline copper electrodes; (**b**) conductive polymer ink; (**c**) conductive textile fabric.

The sensors, front end and power bank (Figure 10a–c) were removed from the neonatal simulation environment and applied to an adult volunteer. The proof of concept trial showed more accurate signal reproduction for the fabricated electrodes compared to the baseline copper electrode than in the mannequin tests. A 15 s sample of each test case is show in Figure 13.

The R peak is visible in each ECG trace having a suitable amplitude for HR calculation, along with the P and T waves. Despite the high pass filtering, baseline wander caused by the EPS susceptibility to movement artefacts (such as respiration) are more prominent in the conductive polymer ink and conductive textile fabric electrode cases.

**Figure 14:**
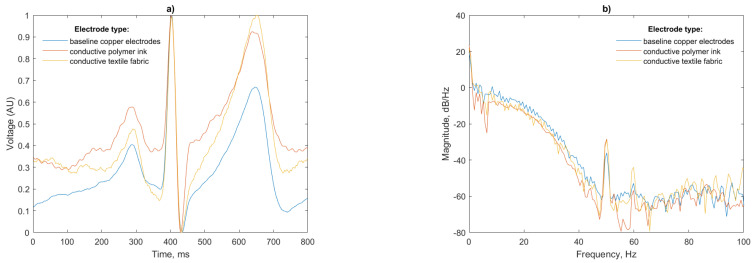
(**a**) Single beat waveform and (**b**) PSD of a human ECG for each electrode configuration.

The power spectral density for the three test cases (Figure 14b) shows that again, as in the mannequin test, the ink and conductive textile fabric electrodes pick up more 50 Hz noise than the copper electrodes. This peak, however, is substantially lower than the peak previously recorded (−30 dB/Hz compared to −10 dB/Hz for the mannequin). The thickness and relative permittivity of the cotton layer, which is not present in the baseline copper electrodes, alters the capacitance of the skin-electrode interface being responsible for this increase in noise in agreement with [35].

Discrepancies in the amplitude of the P and T waves visible in Figure 14a are likely caused by slight differences in the locations of the sensors on the subjects torso from test to test; however, the P, R and T waves are of equal width with matching peaks indicating that the ECG is faithfully reproduced. The peak of the T wave for the conductive textile electrode is as large in magnitude as the R peak for this particular beat; however, this will not affect the R peak detection, as the detection algorithm uses the derivative of the signal. Given this, the gradual slope of the P wave will not be considered as a peak candidate.

### 3.3. Signal Quality Assessment

There is no standardized metric for defining ECG signal quality; therefore, to provide a robust analysis for both the silver conductive and textile conductive electrodes, in comparison with the baseline copper electrodes, three methods have been considered. These are waveform averaging, wavelet-based ECG delineation, relative power in the baseline and the kurtosis of the signal quality indices.

#### 3.3.1. Waveform Averaging

**Figure 15:**
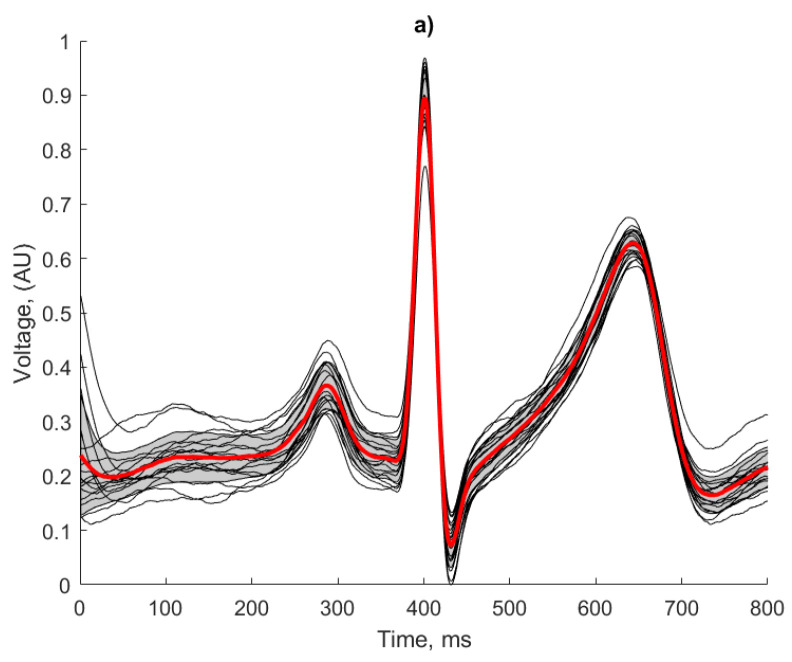

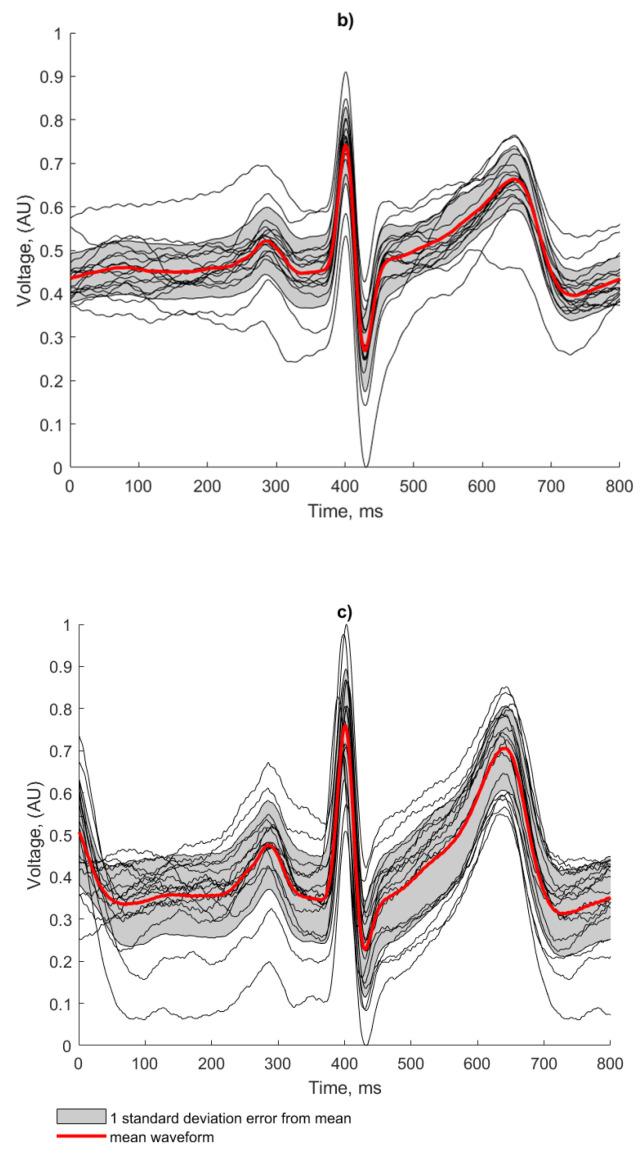
Waveform averaging of the human ECG for (**a**) baseline copper electrodes; (**b**) conductive polymer ink; (**c**) conductive textile fabric.

A waveform averaging approach as described in [36] has been used for signal quality assessment and the identification of heartbeats. Irrespective of the actual morphology of the ECG features for a given signal, this technique considers the regularity of a signal and can be used as a strong indicator of the repeatability and reliability of the ECG recording method, as any artefacts introduced in the recording or processing would result in morphological irregularities. The following approach was applied in the human ECG recordings. The results of this analysis are presented in Figure 15 and Table 2:

1. R-peaks were detected using the Pan-Tompkins algorithm and an average R-R interval was calculated.
2. Individual heart beats were extracted using a window with a number of samples equal to that of the average R-R interval and centred on each detected R peak.
3. An average of the extracted heart beats was calculated, which was used to derive the correlation coefficient of each beat using the following formula:

(3) $$CorrXY=\frac{\sum_{i=1}^{n_{window}}\left(x\left(i\right)-\mu_{x}\right)\cdot \left(y\left(i\right)-\mu_{y}\right)}{\sqrt{\sum_{i=1}^{n_{window}}{\left(x\left(i\right)-\mu_{x}\right)}^{2}}\sqrt{\sum_{i=1}^{n_{window}}{\left(y\left(i\right)-\mu_{y}\right)}^{2}}}$$

where *n_window_* is the number of samples in the individual beat window (in this case 800), *x(i)* is the ECG signal, *y(i)* is the average heartbeat signal and *µ_x_* and *µ_y_* are the means of *x(i)* and *y(i)*. An average of these correlation co-efficients (*µ_CorrXY_*) was taken to obtain a total average correlation coefficient for each test case.

The regularity of the waveforms in Figure 15a show that the copper electrodes provide a reliable baseline for comparison using the manufactured electrodes, with 80% of the waveforms residing within 1 standard deviation of the mean. This is evidenced by the high correlation co-efficient displayed in Table 2.

Both fabricated electrodes (Figure 15b,c) show the P and T waves broadly represented correctly in terms of peak position and width. The R peaks were correctly identified for all beats by the Pan Tompkins algorithm, but there is more variation than in the baseline case. The conductive polymer ink electrodes (Figure 15c) showed the worst beat to beat signal reproduction, with the lowest correlation co-efficient and clearly deformed features of the ECG. Despite the effects of baseline wander affecting the amplitude of the signal, the conductive textile fabric electrodes produced regular and repeatable waveforms, with only 25% of the beats falling outside one standard deviation from the mean. This observation is confirmed by the higher correlation coefficient score obtained by the conductive textile fabric electrodes in comparison to the conductive polymer ink electrodes (see Table 2).

#### 3.3.2. Wavelet-Based ECG Delineation

**Figure 16:**
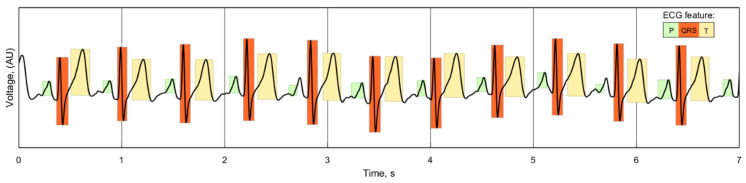
Wavelet delineation of a 7 s sample of recorded ECG, showing the location and duration of the waveform features.

**Figure 17:**
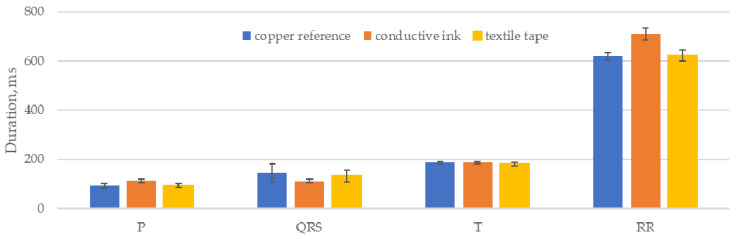
mean P QRS and T lengths and RR intervals for each electrode case.

While there is no standardized consensus on delineating the location of the components of the ECG wave, the repeatability and similarity of individual ECG readings for each of the electrode cases can still be compared. The duration of the P, QRS and T waves of the ECG can be used as an indicator of the signal quality of the reading, as any unwanted noise or phase shift introduced by the electrodes would alter the length and location of these features. A wavelet-based delineation algorithm [37] separates and identifies the features of the ECG, as demonstrated with one of the recorded signals in Figure 16:

This algorithm was implemented in MatLab using the PhysioNet ECGKIT toolbox [38] to compare the duration of the ECG features, as well as the RR intervals, for each of the five 60 s recordings for the three electrode cases. The pooled mean for each case is shown in Figure 17, with an error of 1 standard deviation of the individual means:

The calculated P, QRS and T wave lengths are closely grouped, showing that the electrodes did not introduce any major variation into the waveforms that altered the output of the delineation algorithm. The conductive ink electrodes showed the largest deviations, notably a reduction of 23% in the mean length of the QRS feature compared to the copper reference electrode. Small variations in the feature lengths can be attributed to small variations in the placement of the sensors for each data set but are broadly compensated for by the sample size.

#### 3.3.3. Relative Power in the Baseline and the Kurtosis of the Signal Quality Indices

Two additional signal quality indicators (SQI) are considered for evaluating the proposed electrodes, the relative power in the baseline (pSQI) and the kurtosis of the signal (kSQI). These SQIs are used as indicators of the clinical acceptability of ECGs for HR calculation and further interpretation [39].

pSQI considers the fact that the energy of the QRS feature is concentrated between 5 and 15 Hz [40] and the QRS feature is the main feature used in determining HR; therefore, a comparison of the ratio of power in this band with the band power of the total signal provides a quantifiable measure of ECG quality. pSQI is defined as:

(4) $$\mathrm{baseline}\;\mathrm{relative}\;\mathrm{power},\;\mathrm{pSQI}=\frac{\int_{f=5\;\mathrm{Hz}}^{f=15\;\mathrm{Hz}}P\;\left(f\right)df}{\int_{f=5\;\mathrm{Hz}}^{f=40\;\mathrm{Hz}}P\left(f\right)df}$$

Low pSQI is an indicator of high frequency noise in the ECG, such as the electric potential generated by muscle cells, whereas high pSQI is the result of artefacts such as electrode motion due to subject movement. pSQI values should be within a range of 0.5—0.8 for clinically acceptable ECG recordings [41].

kSQI is a measure of the correlation of the ECG signal. The central limit theorem shows that random signals such as noise tend towards gaussian distributions and correlated signals, such as the repeating pattern of an ECG, tend towards non-gaussian distributions. kSQI is a simple measure of how gaussian-like a signal appears to be, as defined by:

(5) $$\mathrm{kurtosis},\;\mathrm{kSQI}=\frac{E\{{\left(x-\mu_{x}\right)}^{4}\}}{\sigma^{4}}$$

A clean ECG signal generally has a kurtosis of more than 5 and artefacts such as baseline wander and power line interference reduce the overall kSQI [42]. The kSQI was measured for 10 s segments of the ECG and average across all 5 readings.

From the results in Table 3 we can see that all three cases displayed a high pSQI, within the bounds of clinically acceptable ECGs but towards the upper limit. This suggests that electrode motion is present in these recordings, which is clear from the baseline wander shown in Figure 13b,c where it is clearly more pronounced in the textile-based electrodes than in the copper reference electrodes.

The kSQI results clearly show the influence of 50 Hz noise in the recordings (also visible in the noise peaks in the power spectral density in Figure 14b) with a reduction of 22% and 15%, respectively, for the conductive ink and textile fabric electrode in comparison with the reference case.

The waveform averaging, wavelet-based delineation and signal quality indices all show strong correlation between the baseline copper electrodes and the textile-based electrodes. In particular the main feature of interest (the R peak) is reproduced allowing for accurate HR calculation in both textile-based electrodes.

#### 3.3.4. Heart Rate Acquisition Time

**Figure 18:**
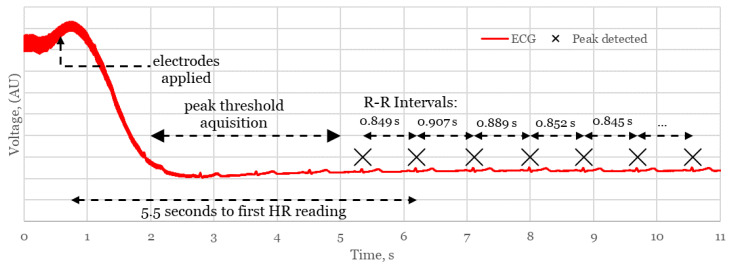
Connection event showing time from sensor application to first HR measurement.

One of the primary requirements of a device for monitoring during resuscitation is that HR readings should be displayed within a time frame below 30 s to avoid oxygen deprivation consequences, such as cerebral palsy and impaired motor skills. In this section, one of the main advantages of the proposed system is the reduced time from birth to HR measurement. This is assessed in Figure 18 which shows a typical connection event from the proof of concept trial considering the adult participant:

The system takes ~5.5 s to register an RR interval, after a 3 s delay for the Pan Tompkins algorithm to establish a threshold for R peaks. An averaged heart rate is compiled from the subsequent measured RR intervals to generate a stable reading in situations where there may be a loss of connection or incorrectly registered peaks in the presence of noise. Different competing systems use various sizes of window to generate the HR, dependent on technology and manufacturer. In our case, a stable and accurate heart rate was consistently measured using a window of 5 RR intervals, suggesting a minimum time from application to HR of around 8 s for a typical neonate with a HR of 140 bpm. This confirms the potential of the device to compete with and surpass, existing delivery room technologies in rapidity of HR measurement.

## 4. Discussion and Conclusions

This work showed that the complete prototype system with novel EPS sensors and textile-based electrodes successfully recorded electrocardiograms from both a simulated and human source. Signal conditioning and filtering in the analogue and digital stages removed unwanted noise and produced a high-quality ECG suitable for heart rate measurement.

Both fabricated silver and conductive fabric electrodes accurately reproduced the R peak timing of the simulated ECG signal to ~0.5% of the baseline copper electrodes. The human ECG was also successfully reproduced using the textile based electrodes and in comparison with the baseline copper electrodes, where the temporal features of the ECG (QRS complex and P T wave) were correctly located and the width of the QRS complex was within 1 ms of the copper reference electrode for both cases. Both implementations of textile-based electrode generated reliable and repeatable signals, with correlation coefficients of 0.90 and 0.87 for the conductive textile fabric and conductive silver ink electrodes, respectively, compared to 0.99 for the reference copper electrode. The regularity of the signal acquired from the textile-based electrodes combined with their high signal quality gave a 100% detection confidence for each of the five 60 s human recordings, confirming their suitability to be used for ECG recordings.

A foam pad was used in the human trial to ensure both sensors were located at a repeatable position; however, small deviations in body and sensor position translated to amplitude changes in the ECG waveform which limited the amplitude feature analysis in comparison to the baseline copper electrode ECG features. Further investigations could be performed using a range of textile-based electrodes simultaneously to investigate amplitude effects, or in simultaneous comparison with a traditional AgCl electrode to determine any RR interval variations induced by different electrode configurations.

In comparison with the reference copper electrodes, the larger surface area and materials of the fabricated textile-based electrodes are more prone to picking up ambient 50 Hz noise. Therefore, the conductive polymer ink and conductive textile fabric electrodes collected larger amounts of 50 Hz noise added to the resultant signal of the human ECG by 30% (−40 dB compared with −30dB for the copper electrodes), where the output signal contained visible 50 Hz noise despite large amounts of frequency specific filtering capacity. The resultant reduction in signal to noise ratio is in part due to the sensing of the ECG through a layer of cotton fabric as well as the alteration of the skin-electrode capacitance and the electrodes being prone to receive ambient 50 Hz noise. Despite this increased noise level, both test cases still produced a signal of sufficient quality for the calculation of HR from RR interval data.

The prototype system described here uses two electrodes. Future work on the prototype system could involve increasing the number of sensors to reduce the 50 Hz noise and providing a greater contact area for readings for the case when the neonate is moved during resuscitation efforts.

This work provides a strong proof of concept for the embedding of textile-based electrodes in a delivery room mattress for ECG measurement, as well as the demonstration of a complete standalone system for HR assessment, data acquisition and display. The extensive characterisation of conductive textile fabric and conductive silver ink electrodes in comparison with a standard reference copper electrode confirms their suitability for use in such a device. These novel flexible electrodes are suitable for embedding in a delivery room mattress and provide the benefits of conformity for increased signal quality and a reduction in the chance of abrasive damage to delicate newborn skin. Signal quality assessment confirmed that high quality ECG recording was achieved in the presence of interference layers and an air gap, with reliable and repeatable ECG waveforms. The device described here could conceivably be used to automatically acquire the HR of a newborn immediately after delivery without the need to attach additional sensors in a safe, rapid and reliable way.

## Figures and Tables

**Table 1:** RR interval comparison of the mannequin simulated signal for each electrode case for a 60 bpm and 140 bpm generated test signal.

	Mean RR Interval, ms		Average bpm	
Electrode:	60 bpm	140 bpm	60 bpm	140 bpm
baseline copper	999	428.6	60.1	140.2
conductive polymer ink	1001	431.6	59.9	139.0
conductive textile fabric	1004	428.3	59.8	140.0

**Table 2:** Average correlation coefficients.

Electrode Type	Average Correlation Co-Efficient, *µ_CorrXY_*:
baseline copper electrodes	0.987
conductive polymer ink	0.865
conductive textile fabric	0.902

**Table 3:** Signal quality indices for each of the electrode cases.

Electrode:	pSQI	kSQI
baseline copper	0.79	4.9
conductive polymer ink	0.74	3.8
conductive textile fabric	0.74	4.2

## Data Availability

The data presented in this study are available on request from the corresponding author. The data are not publicly available due to biometric identity information.
